# Estimating the Outcomes of Intracerebral Haemorrhage with Intracerebral Haemorrhage Score and Acute Physiology and Chronic Health Evaluation-II Score: A Multicentre Study

**DOI:** 10.5152/TJAR.2022.21422

**Published:** 2022-12-01

**Authors:** Hande Gürbüz, Hülya Topçu

**Affiliations:** 1 Department of Anaesthesiology and Reanimation, Bursa Yüksek İhtisas Training and Research Hospital, Bursa, Turkey; 2 Department of Anaesthesiology and Reanimation, Hitit University, Erol Olçok Training and Research Hospital, Çorum, Turkey

**Keywords:** APACHE, cerebral haemorrhage, mortality, patient outcome assessment, stroke

## Abstract

**Objective:**

Spontaneous intracerebral haemorrhage causes mortality or leads to permanent disability in most of the survivors. Thus, determining the severity of the disease to predict mortality and morbidity is important. This study aimed to evaluate Acute Physiology and Chronic Health Evaluation-II and Intracerebral Haemorrhage scores in spontaneous intracerebral haemorrhage patients treated in intensive care units.

**Methods:**

This multicenter study was conducted in 2 tertiary care hospitals’ general intensive care units. Short- (in-hospital) and long-term (1-year) mortality and functional outcomes at discharge were evaluated using the Intracerebral Haemorrhage and Acute Physiology and Chronic Health Evaluation-II scores.

**Results:**

Of the 35 spontaneous intracerebral haemorrhage patients analysed, the modified Ranking Scale was <4 in 10 (28.6%) patients and ≥4 in 25 (71.4%) patients. The in-hospital mortality was 51.4%, and 1-year mortality was 60%. The discriminative power of Acute Physiology and Chronic Health Evaluation-II was excellent (area under the curve ≥0.9), and Intracerebral Haemorrhage Score was fair (area under the curve ≥0.7) for both in-hospital mortality and poor outcomes at discharge. The area under the curve of Acute Physiology and Chronic Health Evaluation-II was significantly higher than the area under the curve of Intracerebral Haemorrhage score.

**Conclusion:**

Acute Physiology and Chronic Health Evaluation-II score is a better model with high sensitivity and specificity than the Intracerebral Haemorrhage score in predicting the in-hospital mortality and functional outcomes at the discharge of spontaneous intracerebral haemorrhage patients. However, the Acute Physiology and Chronic Health Evaluation-II score lacks the neuroradiologic features that are crucial for spontaneous intracerebral haemorrhage. Therefore, the Intracerebral Haemorrhage score can be used as an indicator of neurological status combined with the Acute Physiology and Chronic Health Evaluation-II score rather than as a predictive model of outcomes.

**Main Points**

- Spontaneous intracerebral haemorrhage (SICH) has been reported to be the most common cause of early death in intensive care units and the most frequent reason for brain death. Accordingly, it becomes important to determine the severity of the disease to predict mortality and morbidity.
- The Acute Physiology and Chronic Health Evaluation-II severity scale is a good predictive model for in-hospital mortality, and it is routinely used in many intensive care units. However, it is not a disease-specific scale and lacks radiological features. It is complex and requires a calculator.
- The Intracerebral Haemorrhage score is the most widely used clinical grading scale specific for SICH; it is easy to remember, easily applicable at the bedside, and does not require a calculator.

## Introduction

Spontaneous intracerebral haemorrhage (SICH) accounts for about 10% of strokes affecting millions of people yearly, yet causing mortality at very significant rates and leading to permanent disability in most of the survivors.^1,2^ The SICH has been reported to be the most common cause of early deaths in intensive care units (ICUs).^3^ Unlike other stroke types, the unfavorable outcomes of SICH have not decreased over time. Additionally, SICH remains the most frequent reason for brain death.^4^ For this reason, it becomes important to determine the severity of the disease to predict mortality and morbidity.

Several SICH-specific scoring systems have been proposed, and additionally, many other severity scales are used in ICUs.^5^ An earlier study evaluated the performance of newly developed intensive care severity scales and SICH-specific scoring systems in predicting short- and long-term mortality of SICH.^6^ Although these newly developed scoring systems have been previously shown to perform well, many are not used in routine daily clinical practice. Therefore, this raised the question of whether the grading scales widely used by clinicians are adequate in predicting the outcomes of SICH patients treated in general ICUs.

The Acute Physiology and Chronic Health Evaluation-II (APACHE-II) severity scale is routinely used in our country by integrating into medical database systems in line with ICU quality standards. However, APACHE-II is not a disease-specific scale, especially radiological findings that directly reflect clinical importance in neurological diseases are not included in the calculation. The Intracerebral Haemorrhage (ICH) score is the most widely used clinical grading scale developed by Hemphill et al^7^ as a communication tool among physicians. However, disease-specific grading scales such as ICH scores are often overlooked in most general ICUs, although their use is recommended. Consequently, this study sought to evaluate ICH and APACHE-II grading scales’ performance for characterising functional outcomes and mortality in SICH patients treated in general ICUs.

## Methods

### Patient Population

After the approval of the Karabük Institutional Review Board (no: #2019/65), this multicenter study was conducted in 2 tertiary care hospitals’ general ICUs and was carried out with the ethical standards outlined in the Helsinki Declaration.

The patients admitted to the ICUs from January 2019 to December 2019 with SICH diagnoses were included in the study. The patients with secondary ICH related to trauma, tumor, and aneurysm were excluded from the study. The requirement for written informed consent was waived by the ethics committee for this historical cohort study.

### Data Collection

Demographic information, medical history, pre-treatment blood pressure levels, electrocardiographic analysis, and laboratory and imaging data were recorded. Hematoma volume was measured using the ABC/2 formula (excluding the intraventricular haemorrhage volume).^8,9^ Lobar and non-lobar localisation of the intracerebral haemorrhage was assessed as previously described.^10^

The National Institutes of Health Stroke Scale (NIHSS) scores were calculated at the time of initial evaluation by the neurologist. The Glasgow Coma Scale (GCS) scores were recorded at the first physical examination in the ICU. The APACHE-II scores were calculated using the hospital’s medical database’s calculator at the end of the first 24 hours in the ICU. The ICH scores were evaluated using the GCS and radiological findings as previously described.

Medical treatments, surgical interventions, and complications during the intensive care period were recorded. Additionally, in-hospital mortality, brain deaths, and discharge information (home or palliative care) were acquired. All the patients were maximally treated till the end. Early care limitation or withdrawal of life support was instituted in none of the patients. The decision of transferring the patient to palliative care included severe disability corresponding to a modified Rankin Scale (mRS) score of 4 or 5. The 1-year survival data were acquired from the Civil Registry Department in December 2020.

### Statistical Analysis

The statistical data were analysed using Statistical Package for the Social Sciences Statistics for Windows version 19.0, 2010 (IBM Corp., Armonk, NY, USA) and MedCalc Statistical Software version 19.8 (MedCalc Software bv, Ostend, Belgium). Non-normally distributed continuous variables according to the Shapiro–Wilk test were analysed with the non-parametric Mann–Whitney *U* test. The significance between the categorical data was analysed with the Pearson chi-square or Fisher’s exact (where appropriate). The receiver operating characteristic curves (ROC) were constructed for in-hospital mortality and poor functional outcomes at discharge. Poor functional outcomes were defined as mRS 4-5 (the need for palliative care) and mRS 6 (death). The APACHE-II and ICH scores’ predictive accuracy was assessed by calculating the area under the curve (AUC) of the ROC curves using the non-parametric method. The Hanley and McNeil test was used to compare the AUC of ICH and APACHE-II scores. The variables were presented as numbers (percent) and median (25-75 percentiles). *P* <.05 was considered statistically significant.

## Results

The study was conducted with 35 SICH patients treated in the general ICUs of 2 different tertiary medical centers between January and December 2019. General characteristics, medical history, and short- and long-term outcomes of the patients are presented in Table 1. After completing the intensive care treatment, 10 (28.6%) patients were discharged home, and 7 (20.0%) patients were transferred to palliative care due to severe disability. The in-hospital mortality of the SICH was recorded as 51.4% (18 patients), and 3 patients died due to any reason within the first year after their discharge from the hospital. The causes of death of 18 patients who died in the hospital were hospital-acquired infection/sepsis in 6 (33.3%) patients, cardiac causes in 5 (27.8%) patients, and medical reasons related to the first neurological injury in 2 (11.1%) patients. Brain death was declared in 5 patients (27.8%), and 2 families accepted organ donation.

The comparisons of the characteristics of survived and non-survived (in-hospital mortality) patients are presented in Table 2. The haemoglobin and haematocrit levels and GCS scores were significantly lower in the non-survivor group than in the survivors. The NIHSS scores, duration of ICU stay, haematoma volumes, and cardiac arrhythmias were significantly higher in the non-survivor group than the survivors. The median (25-75 percentiles) APACHE-II scores were 16.0 (11.5-22.5) in the survived group and 38.5 (32.0-43.5) in the non-survived group. The median (25-75 percentiles) ICH scores were 2.0 (1.0-2.0) in the survived group and 3.0 (2.0-4.0) in the non-survived group.

**Figure 1.**
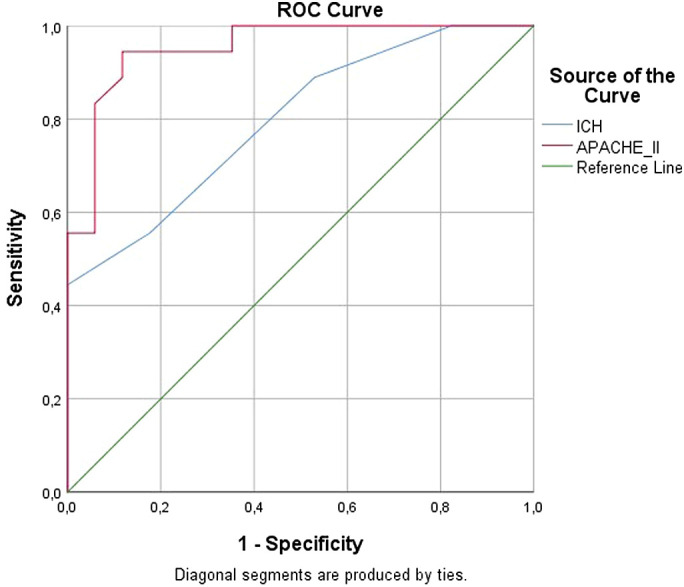
ROC curves for APACHE-II and ICH scores for in-hospital mortality. APACHE, acute physiology and chronic health evaluation; ICH, intracerebral haemorrhage; ROC, receiver operating characteristic.

**Figure 2.**
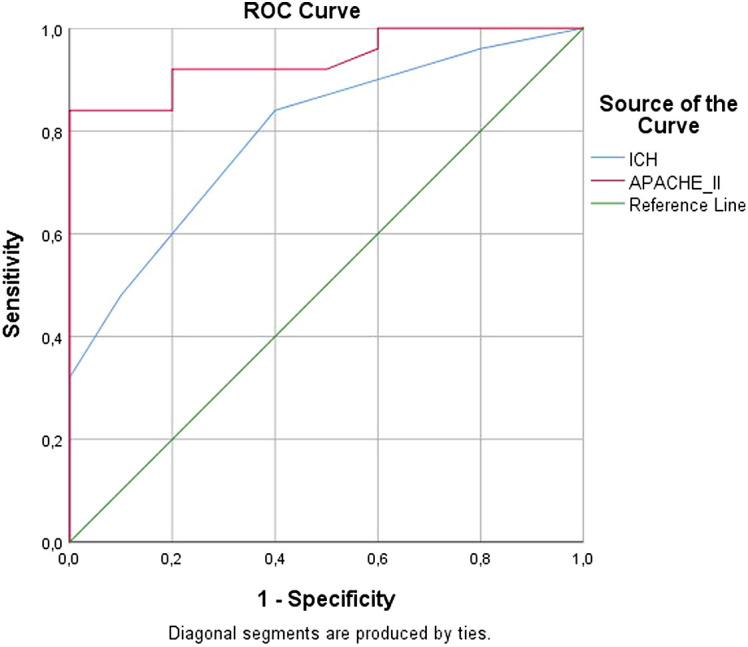
ROC curves for APACHE-II and ICH scores for poor functional outcomes at discharge. APACHE, acute physiology and chronic health evaluation; ICH, intracerebral haemorrhage; ROC, receiver operating characteristic.

**Figure 3.**
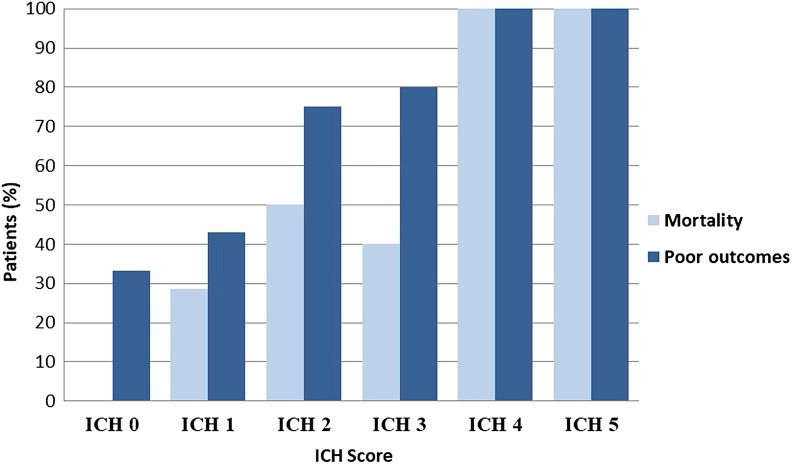
Observed poor outcomes and in-hospital mortality by ICH score ranks. ICH, intracerebral haemorrhage.

The ROC curves drawn for in-hospital mortality and poor functional outcomes at discharge were presented in Figures 1 and  2. The discriminative power of APACHE-II was excellent (AUC ≥0.9), and ICH was fair (AUC ≥0.7) for both in-hospital mortality and poor outcomes at discharge (Table 3). For APACHE-II, sensitivity was calculated as 94.4%, and specificity was calculated as 88.2% with a cut-off value of 24.5 for detecting in-hospital mortality; sensitivity was calculated as 84.0%, and specificity was calculated as 90.0% with a cut-off value of 21.5 for detecting poor outcomes at discharge. For ICH, sensitivity was calculated as 55.6%, and specificity was calculated as 82.4% with a cut-off value of 2.5 for detecting in-hospital mortality; sensitivity was calculated as 84.0%, and specificity was calculated as 60.0% with a cut-off value of 1.5 for detecting poor outcomes at discharge (Table 3). The AUC of APACHE-II score was significantly higher than the AUC of ICH for both in-hospital mortality ( *P*  = .005) and functional outcomes at discharge (*P*  = .002). The observed in-hospital mortality and poor outcome rates for each rank of the ICH score are presented in Figure 3.

## Discussion

The main result of this study is that the APACHE-II score performs as a good predictive model for in-hospital mortality and poor functional outcomes of SICH patients. Although the ICH score’s performance is fair compared to APACHE-II, it has high specificity for mortality and high sensitivity for functional outcomes of SICH patients. Since the ICH score includes components that are not involved in the APACHE-II calculation (such as radiologic features), the ICH score can be used in combination with the APACHE-II to assess SICH patients. Additionally, the ICH score can provide a common language for communication between physicians for SICH patients.

The American Heart Association recommends using a baseline severity score as a part of the initial evaluation of SICH patients.^11^ About 20 disease-specific scoring systems have been produced to determine the prognosis of SICH patients.^12^ But the ICH score is still the most widely used and externally validated SICH-specific score.^13^ Similarly, the APACHE-II score was revised later in a disease-based fashion as the APACHE-IV score by adding various parameters, unfortunately costly. Moreover, the APACHE-IV score was found to have good discriminative power to predict in-hospital and 1-year mortality in patients with SICH.^6^ However, none of these newly developed severity scores were used as widely as APACHE-II in intensive care units.

Only a few studies exist in the literature evaluating the accuracy of APACHE-II in SICH. Huang et al^14^ showed that APACHE-II scores >16 correlated with mortality and poor outcomes in SICH patients. Pan et al^15^ observed that ICH score had better discriminative power for the prediction of 30-day mortality than APACHE-II in SICH. In our study, it was found that the performance of APACHE-II was excellent, and the ICH score’s performance was fair for predicting in-hospital mortality and poor outcomes at discharge. The difference between the results of these 2 studies may be due to the difference in methodology because Pan et al^15^ reported that they calculated APACHE-II scores at the first admission, not at the end of 24 hours, and excluded all patients undergoing surgery and with comorbidity. A previous study mentioned that only 35.7% of in-hospital deaths of SICH patients were due to neurologic criteria; in contrast, most patients died due to their previous comorbidities and hospital-acquired infections/sepsis related to a prolonged hospital stay.^6^ In the present study, we found that 61.1% of in-hospital deaths in SICH patients were related to different reasons other than neurological damage. In addition, considering that most of the SICH patients have at least 1 comorbidity, it is evident that only neurological parameters are not sufficient to predict the outcome of these patients; it also has to be evaluated metabolically.

The APACHE-II score consists of vital parameters in addition to laboratory analysis, mainly showing metabolic impairment. However, the ICH score consists of age, radiological parameters, and GCS, focusing on the neurologic state rather than the metabolic condition. Age, GCS on admission, the haematoma location, haematoma volume, and intraventricular expansion were previously described parameters strongly associated with neurologic deterioration.^16,17^ These parameters, not individually but all together, help to predict the outcomes of SICH patients. Additionally, radiologic findings guide the treatment, for example, a possible need for surgery. None of these parameters that are alone are indicative of the neurological condition. Thus, the ICH score, which includes all these neurologic parameters, can be used as a common communication tool between physicians in SICH patients’ follow-up. Furthermore, the ICH score is easy to remind, easily applicable at the bedside, and does not require a calculator.

None of the severity scores stand alone as an indicator of prognosis. The SICH has often been medically described as a desperate picture, perhaps because it has no specific treatment.^18^ Therefore, it was thought that high severity scores might inadvertently affect physicians, leading to “self-fulfilling prophecies.”^19,20^ It should not be forgotten that the main indicator of prognosis in SICH is medical support and rehabilitation provided.^21^

This study’s limitations are that results might be biased due to the difference in the quality of care in different medical centers. However, since the study is multicentric, its results reflect the general population. Another limitation is that the study is retrospective. Furthermore, previous studies found the presence of intraventricular haemorrhage, haematoma originating from both lobar and non-lobar regions, and infratentorial haemorrhage was (which were not statistically significant in this study) additional independent predictors of mortality in SICH patients. A larger group of patients would also have allowed us to evaluate these parameters. Therefore, while interpreting the results of this study, this factor should be considered.

## Conclusion

Acute Physiology and Chronic Health Evaluation-II score is a better model with high sensitivity and specificity than the ICH score in predicting the in-hospital mortality and functional outcomes at the discharge of SICH patients. However, the APACHE-II score lacks the neuroradiologic features that are crucial for SICH. The ICH score can be used as a communication tool as an indicator of neurological status combined with the APACHE-II score rather than as a predictive model of outcomes.

## Figures and Tables

**Table 1.** Patient Demographics and 1-Year Outcomes

Characteristics	All Patients (n = 35)
Demographics	
Age (years); median (25-75 percentiles)	68.0 (64.0-79.0)
Female; n (%)	18 (51.4)
Male; n (%)	17 (48.6)
Comorbidity	
Hypertension; n (%)	29 (82.9)
Previous cerebral ischemia; n (%)	16 (45.7)
Coronary artery disease; n (%)	10 (28.6)
Diabetes mellitus; n (%)	8 (22.9)
Cognitive dysfunction; n (%)	5 (14.3)
Chronic obstructive pulmonary disease; n (%)	3 (8.6)
Chronic renal failure; n (%)	3 (8.6)
Malignancy; n (%)	3 (8.6)
Thyroid disease; n (%)	1 (2.9)
Previous cerebral haemorrhage; n (%)	1 (2.9)
Drugs	
Acetylsalicylic acid; n (%)	18 (51.4)
Clopidogrel; n (%)	5 (14.3)
Coumadin; n (%)	2 (5.7)
Cardiac interventions/surgery	
Coronary angiography; n (%)	5 (14.3)
Coronary by-pass grafting; n (%)	2 (5.7)
Prosthetic valve replacement; n (%)	1 (2.9)
1-year outcomes	
Discharged; n (%)	17 (48.6)
Home; n (%)	10 (28.6)
Palliative care; n (%)	7 (20.0)
Died; n (%)	21 (60.0)
In-hospital; n (%)	18 (51.4)
Within 1 year; n (%)	3 (8.6)
Brain death	
Declared brain death; n (%)	5 (14.3)
Organ donation; n (%)	2 (5.7)

**Table 2.** Clinical, Laboratory, and Radiologic Findings on Admission According to In-Hospital Mortality. Characteristics of Surgical Treatment and ICU Follow-Up

	Survivors (n = 17)	Non-survivors (n = 18)	*P*
Laboratory findings on admission			
Glucose (mg dL^−1^)	121.0 (102.0-143.5)	180.5 (100.8-250.5)	.094
Haemoglobin (g dL^−1^)	13.9 (12.5-14.9)	11.2 (10.3-13.5)	.006*
Haematocrit (%)	41.4 (38.3-44.3)	34.5 (30.9-39.8)	.003*
Neurologic assessment			
Age (years)	67.0 (64.0-76.5)	70.0 (61.0-80.3)	.851
NIHSS	12.0 (7.0-17.5)	22.0 (16.0-27.0)	.006*
GCS	10.0 (7.5-13.0)	6.0 (3.8-8.3)	.005*
Cardiac assessment			
Systolic blood pressure (mmHg)	200.0 (120.0-235.0)	187.5 (103.8-222.5)	.363
Diastolic blood pressure (mmHg)	100.0 (80.0-117.5)	95.0 (58.8-110.0)	.290
Arrhythmic ECG; n (%)^†^	2 (11.8)	10 (55.6)	.006*
Radiologic assessment			
Location			
Supratentorial; n (%)	16 (94.1)	16 (88.9)	N/A
Infratentorial; n (%)	1 (5.9)	1 (5.6)	
Both supra and infratentorial; n (%)	0 (0.0)	1 (5.6)	
Lobar; n (%)	5 (29.4)	10 (55.6)	.175
Non-lobar; n (%)	11 (64.7)	6 (33.3)	
Both lobar and non-lobar; n (%)	1 (5.9)	2 (11.1)	
Intraventricular haemorrhage; n (%)^†^	5 (29.4)	9 (50.0)	.214
Haematoma volume ≥30 mL; n (%)^†^	6 (35.3)	13 (72.2)	.028*
Surgery			
Non-surgical; n (%)	13 (76.5)	12 (66.7)	.711
Haematoma evacuation and/or decompression; n (%)	4 (23.5)	6 (33.3)	.711
External ventricular drain; n (%)	1 (5.9)	3 (16.7)	.603
Intensive care unit			
Mechanical ventilation in the first 24 hours; n (%)^†^	9 (52.9)	13 (72.2)	.238
Vasopressors in the first 24 hours; n (%)	1 (5.9)	4 (22.2)	.338
Seizures; n (%)	2 (11.8)	0 (0.0)	.229
Length of ICU stay (days)	7.0 (4.5-12.0)	15.5 (9.5-32.8)	.017*

^*^ *P*  < .05; ^†^Pearson chi-square; Median (25-75 percentile).

ECG, electrocardiogram; GCS, Glasgow coma scale; ICU, intensive care unit; NIHSS, National Institutes of Health Stroke Scale.

**Table 3.** ROC Curve Analyses of APACHE-II and ICH Scores

	Cut-off	Sensitivity (%)	Specificity (%)	AUC (95% CI)	*P*	*P* (AUC comparison)
In-hospital mortality						
APACHE-II	24.5	94.4	88.2	0.95 (0.89-1.00)	<.001*	.005*
ICH	2.5	55.6	82.4	0.80 (0.65-0.94)	.003*	
Poor functional outcomes at discharge						
APACHE-II	21.5	84.0	90.0	0.93 (0.85-1.00)	<.001*	.002*
ICH	1.5	84.0	60.0	0.80 (0.64-0.95)	.007*	

^*^ *P*  < .05.

APACHE, acute physiology and chronic health evaluation; ICH, intracerebral haemorrhage; ROC, receiver operating characteristic.
